# An Alternative Model for the Early Peopling of Southern South America Revealed by Analyses of Three Mitochondrial DNA Haplogroups

**DOI:** 10.1371/journal.pone.0043486

**Published:** 2012-09-10

**Authors:** Michelle de Saint Pierre, Claudio M. Bravi, Josefina M. B. Motti, Noriyuki Fuku, Masashi Tanaka, Elena Llop, Sandro L. Bonatto, Mauricio Moraga

**Affiliations:** 1 Instituto de Ecología y Biodiversidad (IEB), Facultad de Ciencias, Universidad de Chile, Santiago, Chile; 2 Programa de Genética Humana, ICBM, Facultad de Medicina, Universidad de Chile, Santiago, Chile; 3 Laboratorio de Genética Molecular Poblacional, Instituto Multidisciplinario de Biología Celular (IMBICE), CCT-CONICET La Plata, La Plata, Argentina; 4 Department of Genomics for Longevity and Health, Tokyo Metropolitan Institute of Gerontology, Itabashi-ku, Tokyo, Japan; 5 Genomic and Molecular Biology Laboratory, Pontifícia Universidade Católica do Rio Grande do Sul, Porto Alegre, Rio Grande do Sul, Brazil; 6 Departamento de Antropología, Facultad de Ciencias Sociales, Universidad de Chile, Santiago, Chile; University of Florence, Italy

## Abstract

After several years of research, there is now a consensus that America was populated from Asia through Beringia, probably at the end of the Pleistocene. But many details such as the timing, route(s), and origin of the first settlers remain uncertain. In the last decade genetic evidence has taken on a major role in elucidating the peopling of the Americas. To study the early peopling of South America, we sequenced the control region of mitochondrial DNA from 300 individuals belonging to indigenous populations of Chile and Argentina, and also obtained seven complete mitochondrial DNA sequences. We identified two novel mtDNA monophyletic clades, preliminarily designated B2l and C1b13, which together with the recently described D1g sub-haplogroup have locally high frequencies and are basically restricted to populations from the extreme south of South America. The estimated ages of D1g and B2l, about ∼15,000 years BP, together with their similar population dynamics and the high haplotype diversity shown by the networks, suggests that they probably appeared soon after the arrival of the first settlers and agrees with the dating of the earliest archaeological sites in South America (Monte Verde, Chile, 14,500 BP). One further sub-haplogroup, D4h3a5, appears to be restricted to Fuegian-Patagonian populations and reinforces our hypothesis of the continuity of the current Patagonian populations with the initial founders. Our results indicate that the extant native populations inhabiting South Chile and Argentina are a group which had a common origin, and suggest a population break between the extreme south of South America and the more northern part of the continent. Thus the early colonization process was not just an expansion from north to south, but also included movements across the Andes.

## Introduction

Reconstruction of the biological history of aboriginal Amerindian populations has been widely debated in the literature for the last two decades. After several years of research, there is now a consensus that America was populated from Siberia (Asia) through the Bering Strait [1], sometime at the end of the Pleistocene. But many details, such as the timing, route, and origin of the first humans, remain uncertain [2].

The extreme south of South America, or Patagonia-Tierra del Fuego (P-TdF), has one of the oldest and most continuous archeological records of early human occupation in the Americas. Monte Verde, in Puerto Mont, Chile, dated at 14,500 years BP [3]–[4] was for a long period of time the oldest archaeological site in America, including North America, the gateway of the first settlers. The Patagonia-Tierra del Fuego region has also many archeological sites with undeniable proof of ancient human occupation. Localities like Cueva Fell (10,000–11,000 years BP), Pali Aike (8,700 BP) [5], Piedra Museo (12,800 BP) [6], and the Tres Arroyos site (11,800 BP) [7] provide evidence of human occupation since at least 12,000 years ago.

In the last decade, genetic evidence has taken on a major role in our knowledge of the peopling of the Americas. One of the markers most extensively used, mitochondrial DNA (mtDNA), has allowed the maternal ancestry of Native Americans to be traced to four major pan-continental haplogroups A-D and one minor North American haplogroup X [8]–[12]. The classical allotment of Native American maternal lineages to haplogroups A-D began to gain better resolution with acknowledgment of the existence of different ethnically/geographically structured founder haplotypes within at least some of the haplogroups [13], [14]. Beginning with the pivotal work of Bandelt et al. (2003) [15], several studies have increased the amount of high-resolution data available, mostly in the form of complete mtDNA sequences and/or complete control region sequences and selected SNP typing. The present landscape of extant Native American mtDNA phyletic diversity is composed of the same major five basal haplogroups A-D plus X, but we are now able to distinguish one to four founder sequences in each haplogroup, adding up to ten monophyletic sub-haplogroups; A2, B2, C1b, C1c, C1d, C4c, D1, D2a, D3 and D4h3a 15–21

In spite of the importance of the P-TdF region, few studies of mitochondrial DNA have been performed with current or historical indigenous populations of the area like the Mapuche, Pehuenche, Huilliche, Yámana and Kawésqar [9]–[10], [22]–[27]. These studies show a cline north to south for the B2, C1b and D1 haplogroups, with B2 decreasing in frequency until it completely disappears in the extreme South in populations such as the Yámana and Kawésqar. C1d and D1 increase their frequencies as we move southward [23]–[27]. Regarding the D-loop sequences of mtDNA, these populations have shown a high frequency of a geographically-linked D1 haplogroup lineage characterized by the presence of the C16187T polymorphism (D1g, according to a report by Bodner et al., 2012 [28]), which was recognized early as specific to the region by Forster et al. (1996) [13]. Genetic studies of populations from the rest of South America appear to confirm this observation, since up until now this lineage has not been found in other Native populations from South America [29]–[51].

In this study, we sequenced the control region of mitochondrial DNA from 300 individuals belonging to indigenous populations of Chile and Argentina and obtained seven complete mitochondrial sequences, to allow a better understanding of the peopling of South America's Southern Cone. Our analyses confirm that sub-haplogroup D1g is a major lineage of the native Patagonians and Fuegians of Chile and Argentina, which would have appeared soon after the colonization of this area. We also found a high frequency of two other mitochondrial lineages characteristic of the region for the B2 and C1b haplogroups, identified by the transitions A470G and C258T, respectively, and preliminarily designated as B2l and C1b13. The haplotype networks of D1g and B2l indicate a high diversity, concordant with the calculation of the Time of Most Recent Common Ancestor (TMRCA) for the two lineages, and suggesting that the current inhabitants are probably descendants of the first colonizers. One further sub-haplogroup, D4h3a5, also appears to be restricted to P-TdF populations. The fact that these lineages are restricted to a specific geographical area has allowed us to elaborate in greater detail the dynamics of the populations that carry them, and thus reconstruct the micro-evolutionary history of southern South America.

## Results

### Mitochondrial lineages

**Figure 1:**
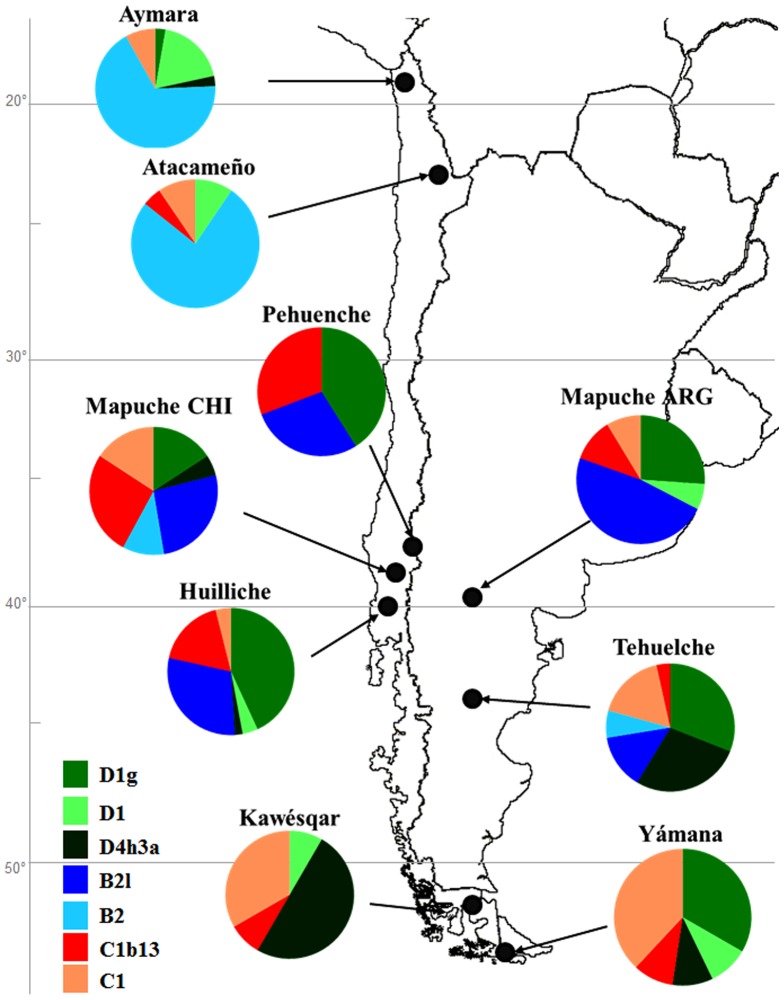
Haplogroup linage map for each population studied. Haplogroup A2 is not included, due to its low representation in these populations. Note the large differences in frequency for D1g, B2l and C1b13 between northern and southern populations.

We analyzed the sequences of the mtDNA control region (rCRS (revised Cambridge Reference Sequence) positions 16032–16544 and 051–555) of 301 individuals belonging to indigenous groups from Chile and Argentina: Aymara, Atacameño, Pehuenche, Mapuche of Chile and Argentina, Huilliche, Tehuelche, Kawésqar and Yámana (see Figure 1 and Table S1). All individuals analyzed were assigned to the American haplogroups A2, B2, C1b, C1c, C1d, D1 and D4h3a, except for one Huilliche assigned to L2a, who was excluded from the analyses (more information on Amerindian haplotypes in Table S5.). Overall, haplogroups B2, C1b and D1 were the most represented; meanwhile low frequencies of A2, C1c and C1d were found in most of the populations analyzed, except for 25% A2 in the Atacameño. B2 peaked in the Atacameño and Aymara, with frequencies >57%, in agreement with values reported for other southern Central Andean populations [22], [29]–[30], [32], [35], [38]–[39], [48]. Consistent with previous reports of both extant and ancient DNA studies in southern South America [9]–[10], [24], [25]–[26], B2 showed a latitudinal clinal variation with higher frequencies in northern Patagonia, intermediate values in Tehuelche and complete absence in the southernmost Yámana and Kawésqar [26]. Frequencies of C1b and D1 also showed a clinal variation, with minor values for the Aymara and Atacameño in the north with respect to southern populations. One Huilliche carried the single C1c lineage reported in this study, while one Kawésqar and one Argentinean Mapuche shared an almost identical C1d sequence attributable to the Patagonian-specific C1d1e branch [52]. The distribution of haplogroup D4h3a was also skewed, being present in only four out of 237 individuals in southern Central Andean and northern Patagonian populations, but accounting for 25% of the 63 southern Fuegian-Patagonian individuals, with values ranging between 10% and 46% in the Tehuelche, Yámana and Kawésqar. All P-TdF individuals belonged to haplogroup D4h3a5 as redefined by us (see nomenclature).

#### Haplogroup D1

**Figure 2:**
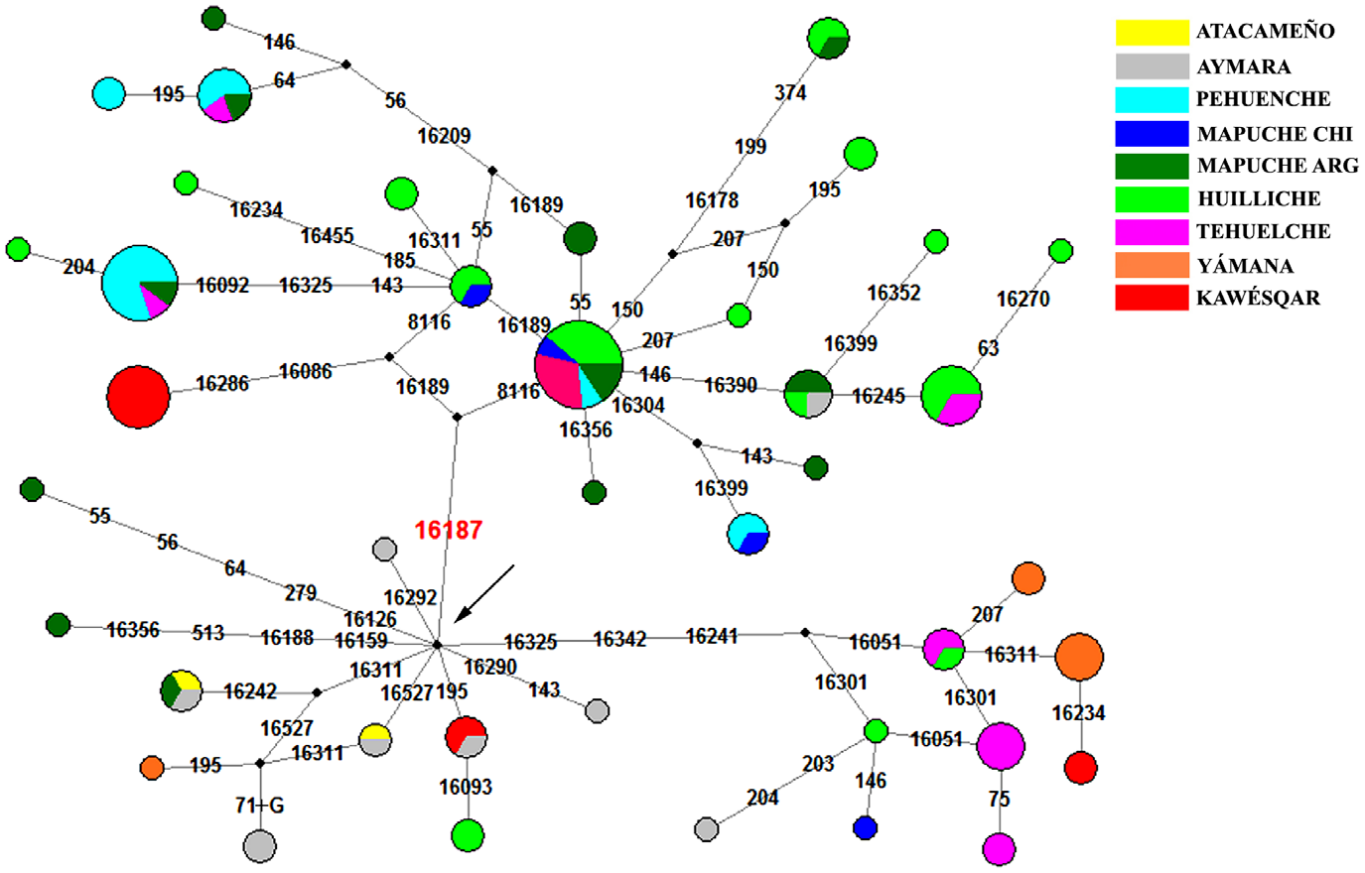
Network for the D haplogroup. The arrow shows the D1 nodal, characterized by rCRS differences at 16223-16325-16362-073-263-315+C-489. Besides the D1 haplotypes, the new lineage D1g is represented here, characterized by the mutation 16187T plus D1 core, and the D4h3a lineages, characterized by 16342 and 16241 polymorphisms.

D1 lineages accounted for 87 out of the 300 sequences in this study (29%), with 70 of them (80%) carrying C16187T, a diagnostic marker of D1g. Clade D1g had a structured pattern of geographic distribution, being found almost exclusively in the central-southern part of Chile and Argentina (Figure 1). In northern Chile only one Aymara had C16187T, sharing a haplotype with one Huilliche and two Argentinean Mapuche (Figure 2), a finding that may be explained by rather recent migratory events. The most southerly record of D1g is at 57°S, where six Yámana shared a haplotype absent in all other individuals analyzed here. Complete mitochondrial sequences were obtained for three D1g individuals (two Tehuelche and one Yámana); we constructed a revisited phylogeny adding our tree sequences to 23 already published by Bodner et al. (2012) [28] (see Figure S1). We confirm the Yámana is the single representative of a new sub-haplogroup called D1g2, not represented in Bodner's phylogeny; the other two samples belong to another sub-haplogroup, D1g1, defined by the coding polymorphism 8116. In order to explore the distribution of these two sub-haplogroups in our sample, we sequenced some additional samples for coding polymorphisms in D1g2 (10202, 10724 and 13020) and D1g1 (8116) (see Table S5). We didn't find any D1g2 polymorphism in any individual analyzed; all belonged to D1g1. We typed the 8116 mutation by PCR-RFLP for the samples not sequenced. Our results indicate that 8116 was absent in the rest of the Yámana, but was present in all remaining D1g samples analyzed.

None of the 13 Kawésqar analyzed here was assignable to D1g. However, the ancient DNA study of García-Bour et al. (2004) [26] described four Patagonia-Tierra del Fuego individuals as D1g, so the presence of this lineage in Kawésqar cannot be ruled out. Since the populations which inhabited the southern extreme of Patagonia have nearly or completely disappeared, it will be hard to determine the real extent of this lineage in southern Patagonia. These can explain the extreme differences in the two clades for D1g; D1g2 was found only in the Yámana and is probably a specific haplotype for the extreme south Patagonian populations, versus the highly diverse D1g1, with six different clusters and present in the remainder populations. Recently, a haplotype D1g1 defined by transitions at 16189, 16209, 55 and 56, and present in the Pehuenche, Argentine Mapuche and Tehuelche, was recovered in five out of seven D1g individuals from the Salitroso Basin (47° 25′S, 71° 29′W), a low-altitude lacustrine basin situated between 100 and 300 m.a.s.l. in central Patagonia, in samples dated between 418±40 and 1,142±42 yr BP (Moraga, personal communication).

#### Haplogroup B2

B2 sequences were found in 34% of the 300 individuals studied. Network analysis showed the presence of three major sub-haplogroups with disjointed geographic distribution. Two of them, defined by mutations T146C-A215G-455+T and C16188T, accounted for two thirds of the B2 lineages in the Aymara and Atacameño. Sequences attributable to these groups have already been reported in both ancient and extant southern Central Andean populations [29]–[30], [32]–[33], [35], [39]–[40], [44], [48]. The third cluster, defined by the presence of A470G, which is completely absent in northern Chile, grouped together 57 of the 61 B2 lineages from Patagonia. We provisionally designated this branch as B2l, but further complete mtDNA sequences are needed in order to describe the lineage properly.

**Figure 3:**
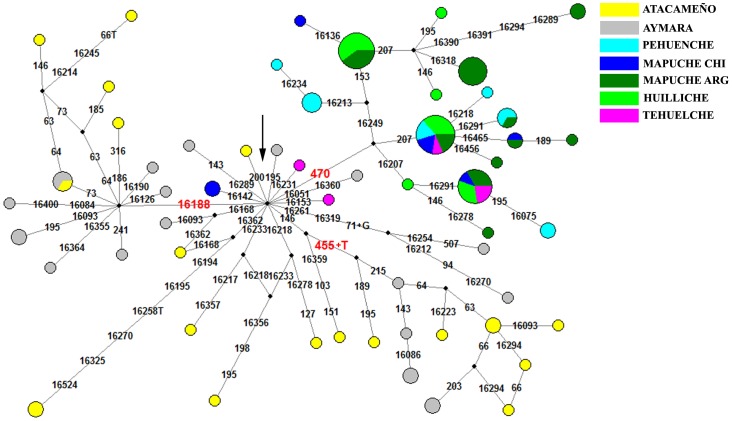
Network for the B2 haplogroup. The arrow shows the B2 nodal, characterized by rCRS differences at 16183C-16189-16217-073-263-315+C-499. We show also the new lineage B2l, characterized by the B2 core plus the 470 polymorphism. The northern haplotypes characterized by the 16188 polymorphism and the 455+T insertion are also noted.

Close inspection of the network encompassing the B2l lineages (Figure 3) shows high variability, which is confirmed by the values of nucleotide diversity (Table S2). There is not a clear geographic structure within clade B2l, to which all individuals from southern Chile belong with the exception of two Mapuche and two Tehuelche. All the evidence suggests that lineage B2l arose early from the B2 haplogroups brought by the early colonizers in the Pleistocene-Holocene limit, and that they evolved independently of the B2 lineages highly represented in northern Chile and Argentina.

#### Haplogroup C1

**Figure 4:**
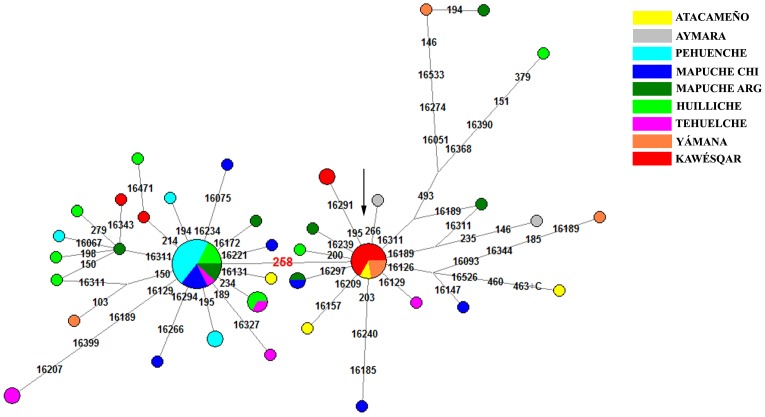
Network for the C1 haplogroup. The arrow shows the C1b nodal, characterized by rCRS differences at 16223-16298-16325-16327-073-249d-263-290d-291d-315+C-489-493-522d-523d. We also show the C1b13 lineage, characterized by the 258 polymorphism plus C1b core.

Sixty-four out of 67 C1 individuals analyzed in this paper were assignable to C1b. Among these, 67% have the polymorphism C258T not previously recognized as a clade in native South Americans, which prompts us to provisionally define this branch as C1b13. Similarly for B2l, complete sequences will be required to confirm this assignment. This clade has greater haplotype but lesser nucleotide diversity than C1 (Table S2), which may be explained by a later appearance of C1b13. The haplotype networks (see Figure 4) of both C1b and C1b13 are star-like, a clear indication of population expansion in recent times, in contrast to the complexity of the networks of D1g and B2l. Branch C1b13 is found mainly between 38° and 42°S (Figure 1), with only a small representation in the Yámana and Kawésqar. The Tehuelche showed a haplotype distribution similar to that found in the Pehuenche, Mapuche and Huilliche. Outside Patagonia, C1b13 lineages were also present in one Atacameño and one Coya from NW Argentina [30].

### Bayesian Analysis

In order to understand the peopling of the extreme south of South America and to date the appearance of the specific lineages mentioned above, we performed analyses with Bayesian statistics using the program BEAST v1.53v. The sequences were grouped using two different criteria, by ethnic affiliation and by phylogenetic affiliation (haplogroups D1g, B2l and C1b13, see Materials and Methods for additional information). For both the construction of Bayesian Skyline Plots and for calculating TMRCA, we used a mutation rate of 30.2% per site per million years from Endicott & Ho, (2008) [53] (see supplementary discussion for the mutational rate choice, Supplemental Discussion S1).

#### Time of the Most Recent Common Ancestor (TMRCA)

To date the appearance of the Patagonian lineages, we combined our sequences with published data of 106 A2, D1, C1 and B2 sequences from South America [30], [54]–[59], generating a data matrix of 406 sequences. The objective was to produce a more complete map of the diversity in South America for the process of dating the specific haplotypes, and thus avoid a possible overestimation of the TMRCA. In Table 1 we show the age of divergence of the lineages D1g, B2l, C1b13 and D4h3a5. Because the discussion about the use of a particular rate is far from resolved, for the TMRCA we decided to compare the ages obtained with different mutation rates, from one considered fast (45%, Howell et al. 2003 [60]) to a slower one (24%, Santos et al. 2005 [61]) and two rates in the mid-range [19]–[53]. We also calculated the rho statistic from the networks for each clade, and used the Soares mutation rate to convert the rho into ages [62]. Besides differences between the ages obtained due to the mutation rates, we found major differences between C1b13 *vs.* D1g and B2l. Taking the mutation rate from Endicott & Ho, (2008) [53] (see Supplemental Discussion S1 and Table S3) we obtained older ages for D1g and B2l (15,175±233 and 14,172±179 years, respectively) than for C1b13; C1b13 was ∼2.5–3 Kyr younger. These ages suggest that at least D1g and B2l originated at the time that the first populations reached the zone, which was at least 14,500 years ago according to the archeological record [3]–[4]. The Howell mutation rate, considered too fast for some authors for settlement studies, gave us a minimum age of ∼10 Kyr for D1g. The C1b13 clade, on the other hand, would have originated somewhat later. The D4h3a5 sub-haplogroup, also mentioned by Perego et al. (2009) [21] but redefined here, has an age of 10,232 yr BP, later than the ages obtained for D1g and B2l, and similar to the one obtained for C1b13. However, because D4h3a5 is found in southern Patagonian populations, principally in the Tehuelche and Kawésqar (Figure 2), it is probable that the rise of D4h3a5 was a local and later event produced in some of the early populations that settled Patagonia.

**Table 1:** Comparison of the coalescence ages calculated using different mutation rates.

Mutational rates/Groups	0.45 m/s/mya^a^ years BP	0.34 m/s/mya^b^ years BP	0.302 m/s/mya^c^ years BP	0.24 m/s/mya^d^ years BP	Soares clock^e^ years BP
**D1g**	10,184	13,479	15,175	19,096	27,174
**B2l**	9,511	12,588	14,172	17,833	22,645
**C1b13**	7,773	10,288	11,583	14,575	14,040
**D4h3a5**	6,867	9,088	10,232	12,875	9,964

m/s/mya = mutation/site/million years ago.

a, b, c & d are calculated based on Bayesian analysis.

e is calculated based on ρ calculation according to Saillard et al., 2000.

a = Howell et al., 2000; b = Kemp et al., 2007; c = Endicott & Ho, 2008; d = Santos et al., 2005; e = Soares et al. (2009).

#### Bayesian Skyline Plots (BSP)

**Figure 5:**
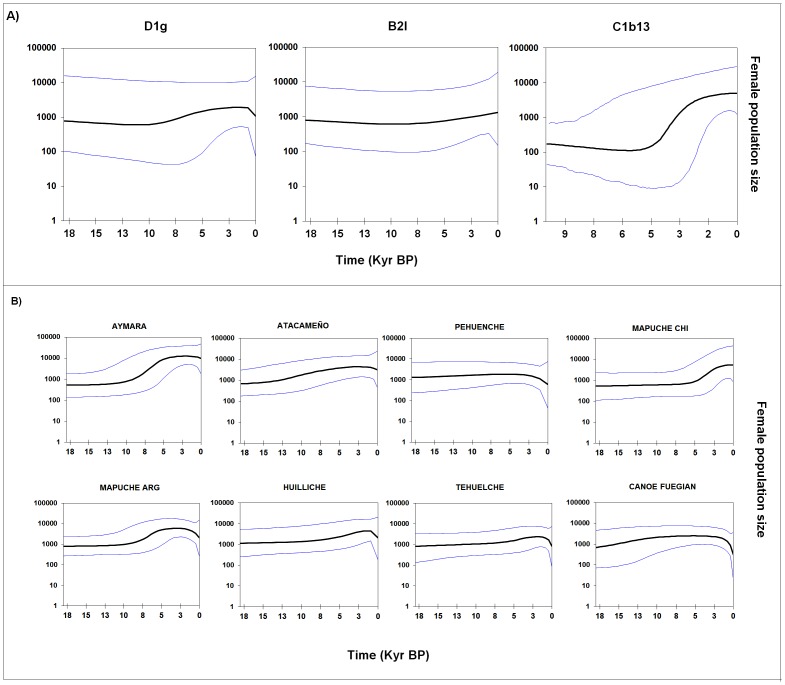
Bayesian Skyline Plot (BSP) showing effective population sizes over time derived from mtDNA D-loop sequences. A) Southern Cone specific haplotypes. B) By ethnic group.

Figure 5a shows the growth curves (Bayesian Skyline Plots, or BSPs) for the specific lineages D1g, B2l and C1b13. The main difference in growth over time is the explosive lineage expansion of C1b13, of an order of magnitude, beginning about 5,000 years BP. For D1g there was constant growth beginning 9,000 years BP, at a slower rate; in contrast the B2l population size remained constant over time, with a slight growth about 3,500 years BP. The great difference in the BSP curve of C1b13 compared to D1g and B2l, added to the differences observed in the haplotype networks and the coalescence age, suggest different events for the emergence of C1b13.

We also performed BSP with the sequences grouped by populations (see Figure 5b). The pattern of growth over time varied among the populations studied, in accordance with their different histories and subsistence strategies. In northern Chile the Aymara showed a large growth, the greatest of the studied populations, beginning around 8,000 BP and reaching its maximum about ∼5,000 years BP, corresponding to the beginning of plant and animal domestication which was the basis of the later development of agriculture [63], and then became stable except for a slight decrease ∼500 years ago, attributable to the catastrophic disruption produced by European colonizers. Despite not having found direct evidence of old agriculture in north Chilean populations, discoveries in Peru shown that the adoption of agricultural techniques by native populations is older than previous estimations. There is evidence of irrigation canals associated with architectural structures dated between 7,600 and 4,500 ^14^C yr BP [64], and also an early adoption of peanut, squash, cotton and quinoa, whose archeological remains have been dated between 9,240 and 5,500 ^14^C yr BP [65]. The curve for the Atacameño, on the other hand, shows a much lower rate of expansion than the Aymara, which may be explained by a greater influence of Andean populations and access to Altiplano's resources for the Aymara than for the Atacameño [54].

In the south, between 38° and 42°S, we analyzed four populations: the Pehuenche, Mapuche (Chile and Argentina) and Huilliche. The BSP graphs show some differences in the population dynamics of these groups. The Mapuche of Argentina and Chile had a similar pattern, with less population expansion than that observed in the Aymara. The Huilliche also showed growth, but less than that of the Mapuche. By contrast, the Pehuenche showed almost no population expansion, clearly different than the Mapuche and Huilliche. It should be noted that the Pehuenche are the only group in which all of the B, C and D lineages belong to clades B2l, C1b13 and D1g (Figure 1). Thus, the differences in the history of their demographic growth may be related to different subsistence strategies, hunter-gatherers in the case of the Pehuenche and agriculture in the case of the Mapuche and Huilliche [66]. Finally, the groups analyzed from southern Tierra del Fuego-Patagonia, the Tehuelche and the “canoe people”, the Yámana and Kawésqar, showed similar historical dynamics with little population growth, which is consistent with their ways of life as terrestrial (Tehuelche) and marine (Yámana and Kawésqar) hunter-gatherers [67]. There was a slight increase in the population size of the Tehuelche in the last 2000 years, which may be due to the movement of the Mapuche to the south, mixing with the inhabitants of southern Patagonia. Complex patterns of interaction between the native inhabitants on both sides of the southern Andes have been recorded in a plethora of archaeological, historical and ethnohistorical studies [68]–[69]. The intensity of contact, with important eastward migrations, increased notably from the 17th century onwards as a strategy to cope with colonial pressure on southern Chilean populations. This process resulted in what is known as the araucanization of Pampa-Patagonia, i.e. the important cultural and biological influence of trans-Andean populations. Further biological admixture occurred as a consequence of the military conquest of Patagonia by the Argentinean Army in the 1870s that resulted in the forced southward relocation of native populations from Northern Patagonia. With regard to the Tehuelche population analyzed in this paper, the available historical and genealogical information both record the recent introgression of both Mapuche and admixed Chileans [70].

### Genetic diversity analyses

We used Wright's F**_ST_** pairwise measure of population differentiation to evaluate the genetic differences among ethnic groups (Table S4), which indicated the existence of two main groups: the populations from northern Chile (the Aymara and Atacameño) and the populations of southern Chile and Argentina, the Huilliche, Pehuenche, Argentine Mapuche and Chilean Mapuche. The Tehuelche, Yámana and Kawésqar were closer to the southern than the northern group; however, they also have high F**_ST_** values with respect to the rest of the southern populations, which indicate that the extreme southern populations remained more isolated yet. A dendrogram constructed from the matrix of F**_ST_** values using neighbor-joining illustrates this situation graphically (Figure S2).

## Discussion

This study analyzed aboriginal populations of Chile and Argentina, some of which are carriers of clades D1g, B2l and C1b13, a set of lineages that so far have been described only in the southernmost part of South America. Their geographic location, diversity and presence in populations confirm that these are three new clades in the Amerindian mitochondrial tree not previously characterized. The estimated ages of D1g and B2l, about ∼15,000 yr BP, together with their similar population dynamics, suggest that they probably appeared soon after the arrival of the first settlers. By contrast, the results for C1b13 show a somewhat later appearance, in a group that underwent a lineage expansion. The differences apparent according to several analyses (network topologies, descriptive statistics, Bayesian Skyline Plot) in C1b13 vs. B2l and D1g also support a different temporal and geographic scenario to explain the origin of D1g and B2l compared to C1b13. A possible scenario to explain the appearance of clades D1g and B2l is that these linages arose in populations of hunter-gatherers in the extreme south with population sizes that were stable for a long period, thus having the time necessary to accumulate mutations and develop more complex patterns, while clade C1b13 originated ∼3–4 Kyr later than D1g and B2l, in a population with increased growth and possibly farther north, where ecological conditions allowed population expansion. The Spanish chronicles of the colonization period for south-central Chile show an extensively populated region, with calculations of over one million inhabitants [71]. This contrasts with the southern region (south of 42°S), where the ecological resources constrained population growth over time, with a calculated population of around 10,000 people for Patagonia-Tierra del Fuego [67].

This analysis also suggests a different microevolutionary history for the north and south. In the north, haplogroup B2 was in greater proportion, 70% in the Aymara and 60% in the Atacameño, and the haplotype diversity was close to 1, the highest found. This, along with the star formation of the networks, the absence of a node and the large number of unique haplotypes both in the Aymara and Atacameño, suggests an important ancient population expansion, estimated to have occurred at least 6,000 years ago by Bayesian analysis. The distribution of the major clades in the Southern Cone did not show large differences among the populations; we did not find clusters linked to a specific population. The principal difference encountered was the high proportion of clade D4h3a5 in southern Patagonia. This clade was originally defined by Perego et al. (2009) [21] but is redefined here (see nomenclature), and is signposted by the presence of 16051 in the control region. D4h3a5 was found exclusively in southern Patagonia-Tierra del Fuego, with the sole exception of one Huilliche. The limited distribution of this lineage reinforces our hypothesis of the continuity of the current Patagonian populations with the initial founders. Additionally, for a specific lineage restricted to the end of the Southern Cone of South America, the dates for D1g and B2l are old and allow us to support a scenario for the settlement of America with dates of the first entry between 18–20 Kyr [17], [54], the first settlers having taken ∼3–5 Kyr to cross the continents and reach Patagonia.

### Origin of the indigenous populations of Patagonia

The majority of the genetic studies in the last few years have focused on the study of mitochondrial lineages, the Y chromosome or autosomes rather than on the phylogeographic relations among Native Americans, which has produced a gap in information on the microevolutionary processes that occurred in South America before the Spanish and Portuguese colonization. Unfortunately, except for the study of the Coya from Argentina [30], none of the available published sequences from indigenous populations of South America has included the complete D-loop; thus there is no information on the SNPs which define C1b13 and/or B2l, making it difficult to compare our results with those of other studies with native populations: the Guaraní, Kaingang [72], Arara [73], Yanomama [74], Zoro, Gaviao and Xavante [51] of Brazil, the Toba, Wichi, Mataco and Pilaga [34] from Argentina, the Ayoreo [36] and Aché [75] of Paraguay, and from the Andean region, the Quechua [32], [35], [39]–[40] and Aymara [29], [32]–[33], [35], [40]. In addition, other studies published in the last three years with complete sequences or at least the D-loop of the mtDNA have been principally focused on the description of Amerindian mitochondrial lineages [17], [21], [52], [54] or in the description of urban South American populations [59], [76]–[81]. From our results and those from the publications mentioned above, it may be inferred there is a complete absence of D1g, C1b13 and B2l in indigenous populations outside of Chile and Argentina. These three lineages, together with the exclusive variants of haplogroup D4h3a, which had a frequency of 87.6% in Patagonia, all suggest strong isolation of the southern populations. We also detected a variant of haplotype B2 in the Aymara (44%) and Atacameño (18.8%) characterized by the polymorphism C16188T; it also has a geographic restriction between 14° and 23°S [29]–[30], [32]–[33], [39], which firms up the north-south disconnection in these two countries. The evident impact the colonization process had, including decimation of entire populations and loss of diversity to different degrees in the remainder, along with relocation, admixture, acculturation, etc. [71], [82], have imposed an extra degree of difficulty on the study of phylogeographic relations, possible colonization routes and demographic processes undergone by Native Americans. Due to lack of data, hypotheses on the peopling, migration routes and origins of Southern Cone of South America populations are scarce.

Recently Rothhammer & Dillehay, (2009) [83], in a revision based on different lines of evidence, proposed two main routes for the peopling of the different areas of South America. From one side, the oldest migration route moved south along the Pacific coast through Chile, following favorable fishing localities and using watercraft, with Monte Verde likely the most southern evidence of this route. The other route could have followed the Andean highlands by way of the river valleys from south to north in Colombia; from there people moved toward the west side of Cordillera, crossing the Altiplano to enter the open parkland country of eastern Brazil and settle the Amazon basin on one side and the Andes of northwest Argentina on the other; from there they spread throughout the Pampas and Patagonia. Therefore, the populations of the two sides of Cordillera in Chile and Argentina should have a closer relationship than those at the same latitude; the populations of Patagonia should be more closely related to those of the Andes of north-west Argentina than with those of southern Chile. Our results indicate, by contrast, the native populations south of 40° S in both Chile and Argentina share a common origin and belong to the same population substrate. We also found no genetic evidence that the migratory route on the oriental side of Andes Cordillera proposed by Rothhammer & Dillehay (2009) would have reached Patagonia. So, Patagonia would have been settled by at least about 15 Kyr ago by migrants that followed the Pacific coast route. Once they arrived in the Monte Verde area, the migrants could not have advanced further along the Pacific coast due to the extension of the glaciers, which covered most of the coast of Patagonia; they crossed the Andes and continued their southern advance on the eastern side. The Andes are low south of 39°S, which would have allowed movement of individuals across them. The whole region would remain isolated afterwards, and the migratory flow would have occurred more in the east-west direction than north-south. Thus the present-day indigenous populations that live south of 38°S are probably descendants of the first settlers, and subsequent migrations from northern regions have had only minor impact on them. They also appear to have remained isolated most of the time, with a minimum migratory flow, probably until the beginning of the Spanish colonization.

## Materials and Methods

### Population Samples

We collected samples from five native populations from Chile and two from Argentina. From northern Chile: (1) Aymara (n = 38), from individuals inhabiting small villages in the Chilean Puna (Putre, Codpa, Esquiña and Illapata, all in the Arica and Parinacota Regions); (2) Atacameño (n = 29), from individuals inhabiting indigenous communities from San Pedro de Atacama, Antofagasta Region. From southern Chile: (3) Mapuche (n = 19) from the city of Temuco, Araucania Region; (4) Huilliche (n = 59), inhabitants of the coastal zone of San Juan, Los Lagos Region; (5) Pehuenche (n = 42) from Trapa-Trapa, (6) Kawésqar (n = 13) from the last descendants of this group, currently inhabiting Punta Arenas, Mallaganes Region, and (8) Yámana (n = 21) from Ukika, Puerto Williams and Isla Navarino. From Argentina: (9) Mapuche (n = 51), from Cerro Policía and Aguada Guzmán, Rio Negro Province, and (10) Tehuelche (n = 29), from Loma Redonda and El Chalía, Chubut Province (see Figure 1 and Table S1 for exact geographic locations).

All samples were from healthy donors from whom informed consent was obtained according to the standards at the time of sampling. Samples were taken at a time when grant institutions for Chile (FONDECYT) did not seek written consent. Oral informed consent was required in all cases. The three projects in which samples were collected, including the oral informed consent protocols, were approved by the ethics committee of the Faculty of Medicine, University of Chile. All data were analyzed anonymously, and only the geographic location and ethnic affiliation of the populations were considered. This study was approved by the Bioethics Committee for Human Research, Facultad de Medicina, Universidad de Chile.

### Molecular analysis

#### Control region sequences

We amplified and sequenced 1016 bp corresponding to the mtDNA control region (rCRS positions 16032–16544 and 051–555) in all the Chilean populations (except for 13 Huilliche, see below). The amplification conditions used were as described elsewhere [26]. The sequencing and purification of the PCR (Polymerase Chain Reaction) products were performed by Macrogen, of South Korea. Sequences were aligned and edited with Alignment Explorer in MEGA 4.0 [84]. Polymorphisms were confirmed directly using Sequencher 4.9 vDemo. Samples from the Tehuelches and Mapuches of Argentina and a fraction of the Huilliches (N = 13) were also PCR-amplified, sequenced and analyzed for the complete control region between positions 16024–576 and the adjacent 5′ portion between positions 15878–16023 in rCRS, following [85]–[86]. An additional quality control check was performed by EMPOP (http://www.empop.org).

#### Analysis of coding region SNPs

Fragments around transitions at 8116, 10202–10724 and 13020 were amplified and sequenced employing primer pairs F7955 (5′-CCCCCATTATTCCTAGAACCA-3′) and B8785 (5′-TCCGAGGAGGTTAGTTGTGG-3′), F10084 (5′-TCAACACCCTCCTAGCCTTA-3′) and B10931 (5′-AGGAAAAGGTTGGGGAACAG-3′), and F12879 (5′-TTTCATCCTCGCCTTAGCAT-3′) and B13590 (5′-CAGGGAGGTAGCGATGAGAG-3′), respectively. Amplicons were sequenced in a set of samples derived from admixed populations in Argentina carrying matches or near matches for the D1g sequences described in this paper and covering all the major subclades recognizable in the network, plus several individuals carrying the nodal haplotype.

All the Chilean D1g samples were analyzed for the presence of 8116 by means of PCR-RFLP analysis. The samples were amplified using the primer pair 8116F (5′-TGAAGCCCCCATTCGTATAA-3′) and 8116R (5′-GTGGGCTCTAGAGGGGGTAG-3′). The 275 bp amplicons produced were digested O.N. at 37°C with the SmaI restriction enzyme. The digestion products were visualized on a 2% Agarose gel.

#### Complete mtDNA sequences

Complete mitochondrial DNA sequences - Korn06, Korn08, Teh14, and Teh50 - were obtained as in Tanaka et al. (2004) [87], while lineages Teh26, Hui28, and YA2D were sequenced following procedures described elsewhere [88]. A phylogenetic tree for haplogroup D was constructed by hand from the complete sequences (Figure S1).

#### Nomenclature

Bodner et al. (2012) defined D1g by the shared presence of transitions at rCRS positions 8116 and 16187. Our findings of Yámana D1 lineages with 16187 but without 8116 led us to propose a revised definition of clade D1g, which is now identified by 16187, while 8116 is restricted to its major nested clade D1g1. A new sister branch that lacks 8116, D1g2, is erected for the Yámana lineages. For the time being, D1g2 remains poorly defined because only one complete mtDNA is available.

In the absence of complete mtDNA sequences that would allow us to establish their deep phylogenetic affinities, we propose to provisionally assign B2l to our Patagonian cluster defined by a control region transition at 470, and assign C1b13 to the clade that includes those lineages carrying 258.

We also propose a revised definition of D4h3a5 (Figure S3). This clade was originally defined by the presence of a back mutation at 16301, a change that is even recurrent for Chilean lineages in the small dataset of 45 complete D4h3a mtDNAs provided by the authors [21]. In our opinion, such a weak definition artificially joined one Patagonian-exclusive cluster with one Peruvian lineage, resulting in a non-monophyletic clade. Further evidence for the artifactual nature of D4h3a5 as defined in [21] stems from the fact that its estimated coalescence age of 25.3–30.6 ky is well beyond the range of ages estimated using similar methods for the other Native American clades by the same authors [21], [51].

### Statistical analysis

#### Summary Statistics

Summary statistics of genetic diversity were calculated using the program Arlequin 3.1 [89] using Tamura-Nei distances [90] and a gamma parameter value of 0.26 [91]. The following summary statistics were computed: total and per population number of segregating sites (S), nucleotide diversity (π), Haplotype diversity (Hd) and mean number of pairwise differences (K). For each analysis, the sequences were grouped by population and haplotype (see Tables S1 and S2). In order to test the demographic structure, we performed Fst analyses in Arlequin ver. 3.1. Sites 309+C and 309+CC were eliminated from all analyses.

#### Haplotype networks

Sequences were grouped by mitochondrial haplogroup (D, B and C) and analyses were performed separately. Sites 16519 and 152 were eliminated due to their homoplasy; the rest of the homoplastic sites were given a low weight in order to avoid non-phylogenetic reticulations. Calculations were made using the Network 4.5.0 program (www.fluxus-engineering.com/sharenet_rn.htm); median joining and maximum parsimony were used as post-processing options.

Supplement networks (A2, B2, C1b and D) were constructed by hand (see Supplemental Networks S1).

#### Demographic reconstruction and age estimation

Bayesian analyses were performed in the BEASTv1.53 program [92]. To study population dynamics over time we generated BSPs, built with the software Tracer v1.5. Sequences were grouped by population and by lineage and analyzed separately, with at least two runs per grouping. Runs used both strict and relaxed log normal molecular clock models; the resulting BSPs were compared. The BSPs were equivalent for all runs with both models except for lineages C1b13 and D1g. For C1b13 it was impossible to construct a BSP with the strict clock; thus we used the relaxed log exponential model. The rate of nucleotide substitution in the runs was 1, which was later corrected using the lower rate of Endicott & Ho, (2008) [53], of 30.2% mutations per million years (see Supplemental Discussion S1 for mutational rate choice). Analyses were run for 100 million iterations, discounting the first 10% as burn-in. Genealogies and model parameters were sampled every 2500 iterations. For the final construction of the BSP, the output from Tracer was corrected for a mutation rate of 3.02E-7 for time and effective population size (Nef). We used the median to calculate the Nef, with a generation time of 25 years. Graphs were constructed in Excel, transforming to log_10_.

To date the appearance of the Patagonian lineages, we added to the studied 107 D-loop sequences other published sequences of A2, D1, C1 and B2 from South America [30], [54]–[59], generating a final data matrix with 406 sequences. The objective was to produce a more complete map of the diversity in South America and thus avoid a possible overestimation of the TMRCA. In the same run we grouped the sequences by lineages, in order to estimate the divergence of each lineage in the same analysis. In order to improve the estimated ages of coalescent ancestors of specific lineages, we calculated rho (ρ) (according to [93]) from the networks (Figure 2, 3 and 4). The ρ values were 3 for D1g, 2.5 for B2l, 1.55 for C1b13 and 1.1 for D4h3a5; these values were transformed to ages with the rho calculator for the control region provided by Soares et al. (2009) (http://www.ajhg.com) [62]. See Table S5 for the dates obtained for each lineage.

### Data access

The GenBank (http://www.ncbi.nlm.nih.gov/genbank) accession numbers for the 287 new D-loop sequences reported in this paper are JQ067699–JQ06794; JQ280314–JQ280336; (pending), and for the seven novel complete mtDNA sequences are (pending).

## Supporting Information

Figure S1 **Phylogenetic tree of southern South American haplogroups D1g.** This tree includes 23 sequences reported by Bodner et al. (2012) and 3 new complete mtDNA sequences, and illustrates subhaplogroup affiliations. The position of the revised Cambridge Reference Sequence (rCRS) (Andrews et al. 1999) is indicated for reading off sequence motifs. All SNPs and indels are shown on the branches except for cytosine insertions at np 309. In the case of transversions, insertions, or heteroplasmic mutations, the base is indicated according to the IUPAC nucleotide code. The prefix@ indicates the reversion of a mutation occurring earlier in the phylogeny. The suffixes “s” and “ns” indicate synonymous and nonsynonymous substitutions, respectively, while “t” and “r” indicate affected positions in tRNA and rRNA loci, respectively. Recurrent mutations within the phylogeny are underlined. The green numbers on this figure are the same used in Bodner's D1g phylogeny figure (Bodner et al., 2012). The red numbers correspond to numbers of the table S6.
(TIF)

Figure S2 **Dendrogram Neighbor Joining built from genetic distances obtained from the pairwise Fst analysis.**
(TIF)

Figure S3 **Phylogenetic tree for D4h3a & D1.** This tree include 7 new complete mtDNA sequences (red numbers), and 7 previously reported (blue numbers, table S6). The position of the revised Cambridge Reference Sequence (rCRS) (Andrews et al. 1999) is indicated for reading off sequence motifs.
(TIF)

Table S1 Geographic location and molecular basic indices of studied populations
(DOC)

Table S2 Molecular basic indices for haplogroup/haplotype lineage.
(DOC)

Table S3 Comparison of ages of TMRCA (years BP) calculated with different mutation rates.
(DOC)

Table S4 Fst values between populations.
(DOC)

Table S5 Sequences of the D-loop region of analyzed populations.
(XLS)

Table S6 Genbank and ID samples for D4h3a and D1 tree.
(DOC)

Supplemental Discussion S1 Discussion about mutational rate used.
(DOC)

Supplemental Networks S1 Network made by hand for mitochondrial haplogroups A2, B2, D1, C1b and D4h3. The numbers correspond to the table S5.
(PPT)
