# Supplementary figures and images for: An Alternative Model for the Early Peopling of Southern South America Revealed by Analyses of Three Mitochondrial DNA Haplogroups
Source: PLoS One. 2012 Sep 10;7(9):e43486. doi: 10.1371/journal.pone.0043486 (PMC3438176; doi:10.1371/journal.pone.0043486)

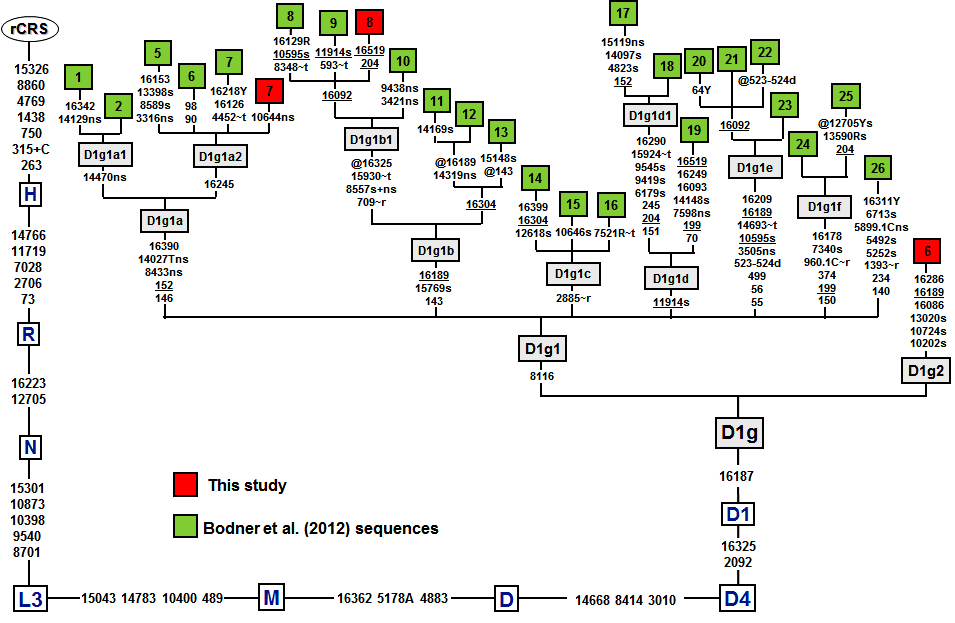

Supplement: Figure S1 — Phylogenetic tree of southern South American haplogroups D1g. This tree includes 23 sequences reported by Bodner et al. (2012) and 3 new complete mtDNA sequences, and illustrates subhaplogroup affiliations. The position of the revised Cambridge Reference Sequence (rCRS) (Andrews et al. 1999) is indicated for reading off sequence motifs. All SNPs and indels are shown on the branches except for cytosine insertions at np 309. In the case of transversions, insertions, or heteroplasmic mutations, the base is indicated according to the IUPAC nucleotide code. The prefix@ indicates the reversion of a mutation occurring earlier in the phylogeny. The suffixes “s” and “ns” indicate synonymous and nonsynonymous substitutions, respectively, while “t” and “r” indicate affected positions in tRNA and rRNA loci, respectively. Recurrent mutations within the phylogeny are underlined. The green numbers on this figure are the same used in Bodner's D1g phylogeny figure (Bodner et al., 2012). The red numbers correspond to numbers of the table S6. (TIF) [file pone.0043486.s001.tif]

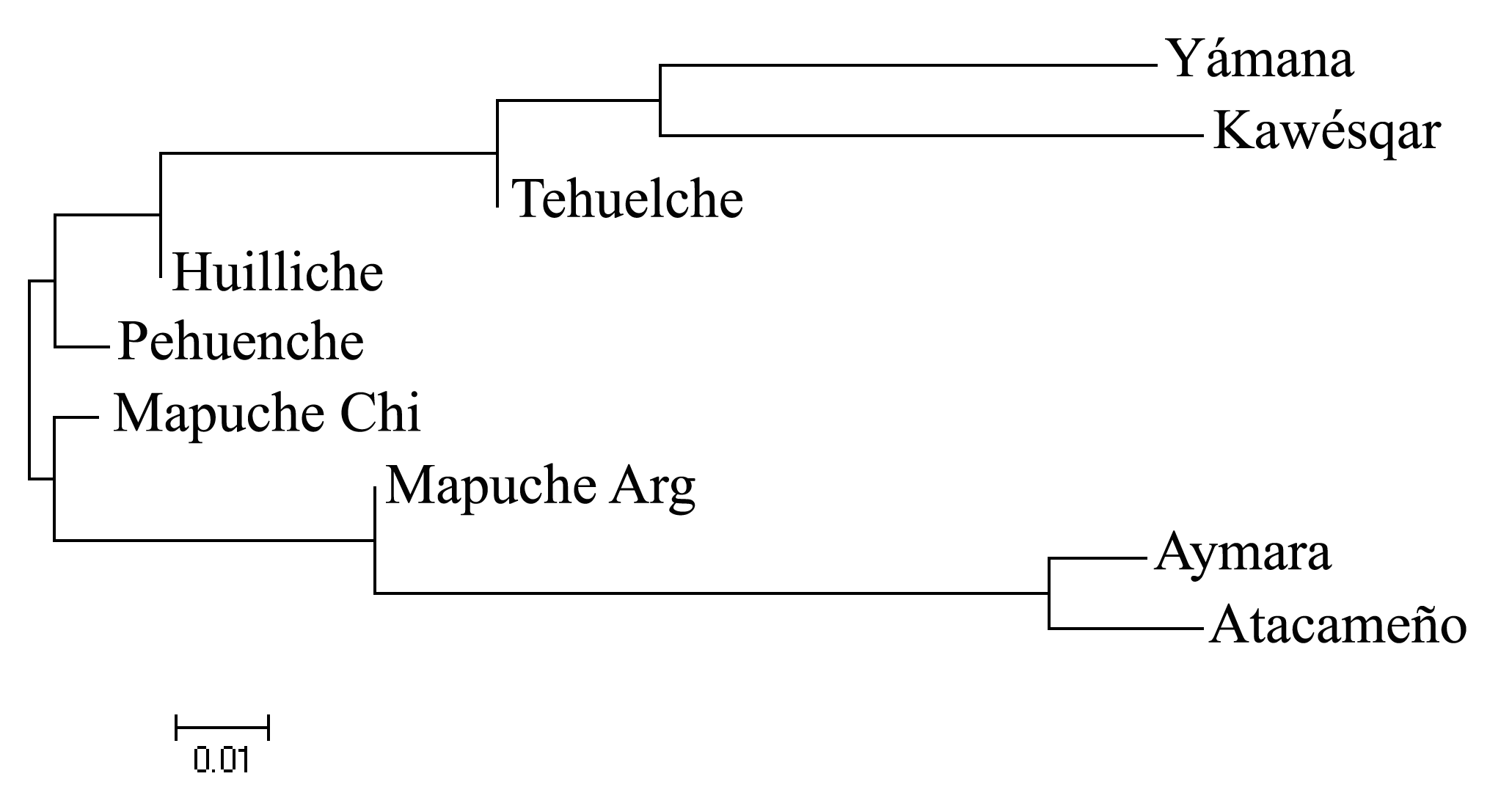

Supplement: Figure S2 — Dendrogram Neighbor Joining built from genetic distances obtained from the pairwise Fst analysis. (TIF) [file pone.0043486.s002.tif]

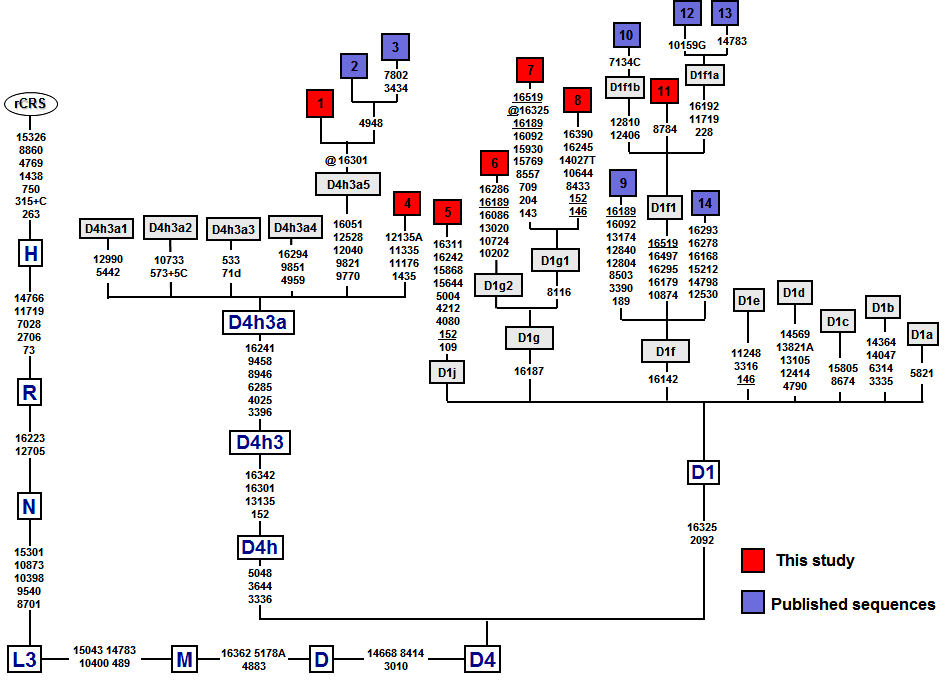

Supplement: Figure S3 — Phylogenetic tree for D4h3a & D1. This tree include 7 new complete mtDNA sequences (red numbers), and 7 previously reported (blue numbers, table S6). The position of the revised Cambridge Reference Sequence (rCRS) (Andrews et al. 1999) is indicated for reading off sequence motifs. (TIF) [file pone.0043486.s003.tif]
