# Supplementary material for: An Alternative Model for the Early Peopling of Southern South America Revealed by Analyses of Three Mitochondrial DNA Haplogroups
Source: PLoS One. 2012 Sep 10;7(9):e43486. doi: 10.1371/journal.pone.0043486 (PMC3438176; doi:10.1371/journal.pone.0043486)
Supplement: Table S1 — Geographic location and molecular basic indices of studied populations (DOC) [file pone.0043486.s004.doc]

**Table S1.** Geographic location of studied populations and molecular basic indices.

|  | Location | n | h | S | Hd | K | π |
| --- | --- | --- | --- | --- | --- | --- | --- |
| Aymara | 17º 22’ S  68º 90’ W | 39 | 34 | 77 | 0.993 | 12.110 | 0.012 |
| Atacameño | 23º 45’ S  68º 17’ W | 28 | 22 | 64 | 0.976 | 14.574 | 0.014 |
| Pehuenche | 37º 43’ S  71º 16’ W | 42 | 17 | 49 | 0.923 | 13.165 | 0.013 |
| Mapuche CHI | 38º 43’ S  73º 36’ W | 19 | 16 | 46 | 0.983 | 12.573 | 0.012 |
| Mapuche ARG | 39º 42’ S  68º 35’ W | 51 | 32 | 72 | 0.973 | 13.999 | 0.014 |
| Huilliche | 40º 50’ S  73º 35’ W | 58 | 32 | 66 | 0.969 | 13.203 | 0.013 |
| Tehuelche | 42º 21’ S  66º 36’ W | 29 | 18 | 50 | 0.961 | 12.217 | 0.012 |
| Yámana | 55º 04’ S  67º 40’ W | 21 | 7 | 26 | 0.814 | 9.438 | 0.009 |
| Kawésqar | 53º 08’ S  70º 55’ W | 13 | 8 | 38 | 0.897 | 12.077 | 0.012 |

n: sample.

h: number of haplotypes.

S: number of polymorphic sites.

H_d_: haplotype diversity.

K: mean number of pairwise differences.

π: nucleotide diversity.
