# Supplementary material for: An Alternative Model for the Early Peopling of Southern South America Revealed by Analyses of Three Mitochondrial DNA Haplogroups
Source: PLoS One. 2012 Sep 10;7(9):e43486. doi: 10.1371/journal.pone.0043486 (PMC3438176; doi:10.1371/journal.pone.0043486)
Supplement: Table S2 — Molecular basic indices for haplogroup/haplotype lineage. (DOC) [file pone.0043486.s005.doc]

**Table S2.** Molecular basic indices for haplogroup/haplotype lineage.

|  | **n** | **H** | **S** | **H_d_** | **K** | **π** |
| --- | --- | --- | --- | --- | --- | --- |
| **D total** | 107 | 44 | 54 | 0.972 | 6.644 | 0.007 |
| **D1g** | 70 | 24 | 34 | 0.944 | 5.118 | 0.005 |
| **D1** | 17 | 11 | 20 | 0.949 | 4.344 | 0.004 |
| **D4h3a5** | 17 | 6 | 6 | 0.868 | 1.885 | 0.002 |
| **C total** | 67 | 40 | 60 | 0.937 | 3.677 | 0.004 |
| **C1b13** | 40 | 22 | 28 | 0.859 | 2.201 | 0.002 |
| **C1b** | 24 | 15 | 28 | 0.891 | 3.358 | 0.003 |
| **B total** | 102 | 57 | 84 | 0.971 | 6.126 | 0.006 |
| **B2l** | 57 | 20 | 25 | 0.913 | 3.895 | 0.004 |
| **B2** | 45 | 37 | 69 | 0.991 | 6.394 | 0.006 |
| **A total** | 24 | 12 | 22 | 0.917 | 4.214 | 0.004 |
| **Total sequences** | 300 | 153 | 161 | 0.999 | 12.882 | 0.013 |

n: sample.

h: number of haplotypes.

S: number of polymorphic sites.

H_d_: haplotype diversity.

K: mean number of pairwise differences.

π: nucleotide diversity.
