# Supplementary material for: An Alternative Model for the Early Peopling of Southern South America Revealed by Analyses of Three Mitochondrial DNA Haplogroups
Source: PLoS One. 2012 Sep 10;7(9):e43486. doi: 10.1371/journal.pone.0043486 (PMC3438176; doi:10.1371/journal.pone.0043486)
Supplement: Table S3 — Comparison of ages of TMRCA (years BP) calculated with different mutation rates. (DOC) [file pone.0043486.s006.doc]

| **Mutational rates/ Groups** | **0.45 m/s/mya^a^ years BP** | **0.34 m/s/mya^b^ years BP** | **0.302 m/s/mya^c^ years BP** | **0.24 m/s/mya^d^ years BP** | **Soares clock^e^ years BP** |
| --- | --- | --- | --- | --- | --- |
| **A2, B2, C1 & D haplogroups** | **28,000** {18.0; 38.9} | **37,059** {23.9; 51.5} | **41,722** {26.9; 57.9} | **52,500** {33.8; 72.9} |  |
| **D1 South America** | **11,800** {7.7; 16.5} | **15,618** {10.2; 21.9} | **17,583** {11.5; 24.7} | **22,125** {14.4; 31.0} |  |
| **D1g** | **10,184** {6.3; 14.8} | **13,479** {8.4; 19.6} | **15,175** {9.4; 22.1} | **19,096** {11.8; 27.8} | **27,174** |
| **D4h3a5** | **6,867** {4.0; 10.0} | **9,088** {5.4; 13.3} | **10,232** {6.0; 14.9} | **12,875** {7.6; 18.8} | **9,964** |
| **B2 South America** | **12,367** {8.5; 16.7} | **16,368** {11.2; 22.1} | **18,427** {12.6; 24.9} | **23,188** {15.9; 31.3} |  |
| **B2l** | **9,511** {6.5; 12.9} | **12,588** {8.6; 17.0} | **14,172** {9.7; 19.2} | **17,833** {12.2; 24.2} | **22,645** |
| **C1 South America** | **11,798** {7.9; 16.3} | **15,615** {10.4; 21.6} | **17,579** {11.7; 24.3} | **22,121** {14.7; 30.6} |  |
| **C1b** | **11,040** {7.6; 14.8} | **14,612** {10.1; 19.6} | **16,450** {11.4; 22.1} | **20,700** {14.3; 27.8} |  |
| **C1b13** | **7,773** {5.3; 10.6} | **10,288** {7.0; 14.0} | **11,583** {7.9; 15.8} | **14,575** {9.9; 19.9} | **14,040** |

**Table S3.** Comparison of ages of TMRCA (years BP) calculated with different mutation rates.

{X;Y} 95% CI (ky**)**, a = Howell et al. (2003), b = Kemp et al. (2007), c = Endicott & Ho, (2008), d = Santos et al. (2005), e = Soares et al. (2009), rho calculation according to Saillard et al. (2000).
