# Supplementary material for: An Alternative Model for the Early Peopling of Southern South America Revealed by Analyses of Three Mitochondrial DNA Haplogroups
Source: PLoS One. 2012 Sep 10;7(9):e43486. doi: 10.1371/journal.pone.0043486 (PMC3438176; doi:10.1371/journal.pone.0043486)
Supplement: Table S4 — Fst values between populations. (DOC) [file pone.0043486.s007.doc]

**Table S4.** Fst values between populations. Pairwise Fst values (below diagonal) and associated P-values (above diagonal). In bold values with statistical significance p<0.05.

|  | **Huilliche** | **Pehuenche** | **Mapuche CHI** | **Mapuche ARG** | **Yámana** | **Tehuelche** | **Kawésqar** | **Aymara** | **Atacameño** |
| --- | --- | --- | --- | --- | --- | --- | --- | --- | --- |
| **Huilliche** |  | **0.048** | 0.094 | 0.068 | **0.001** | **0.028** | **0.003** | **0.000** | **0.000** |
| **Pehuenche** | **0.023** |  | 0.208 | **0.033** | **0.002** | **0.025** | **0.001** | **0.000** | **0.000** |
| **Mapuche CHI** | 0.029 | 0.014 |  | 0.119 | **0.010** | 0.052 | **0.005** | **0.004** | **0.000** |
| **Mapuche ARG** | 0.018 | **0.033** | 0.025 |  | **0.000** | **0.003** | **0.000** | **0.003** | **0.003** |
| **Yámana** | **0.093** | **0.097** | **0.108** | **0.169** |  | **0.002** | **0.019** | **0.000** | **0.000** |
| **Tehuelche** | **0.041** | **0.044** | 0.053 | **0.079** | **0.094** |  | 0.062 | **0.000** | **0.000** |
| **Kawésqar** | **0.106** | **0.140** | **0.138** | **0.163** | **0.112** | 0.052 |  | **0.000** | **0.000** |
| **Aymara** | **0.116** | **0.127** | **0.111** | **0.067** | **0.292** | **0.167** | **0.268** |  | 0.070 |
| **Atacameño** | **0.110** | **0.139** | **0.137** | **0.075** | **0.294** | **0.186** | **0.251** | 0.027 |  |
