# Supplementary material for: An Alternative Model for the Early Peopling of Southern South America Revealed by Analyses of Three Mitochondrial DNA Haplogroups
Source: PLoS One. 2012 Sep 10;7(9):e43486. doi: 10.1371/journal.pone.0043486 (PMC3438176; doi:10.1371/journal.pone.0043486)
Supplement: Table S6 — Genbank and ID samples for D4h3a and D1 tree. (DOC) [file pone.0043486.s009.doc]

| **ID # tree** | **Sample ID** | **GenBank ID** | **Reference** |
| --- | --- | --- | --- |
| **1** | Tehuel 14 | pending | This study |
| **2** | S-973354 | FJ168741 | Perego et al., 2009 |
| **3** | S-686678 | FJ168740 | Perego et al., 2009 |
| **4** | Huilli 28 | pending | This study |
| **5** | Korn08 | pending | This study |
| **6** | YA2D | pending | This study |
| **7** | Teh 50 | pending | This study |
| **8** | Teh 26 | pending | This study |
| **9** | Tor25 | EU431089 | Achilli et al., 2008 |
| **10** | TYR16 | EU095241 | Fagundes et al., 2008 |
| **11** | Korn06 | pending | This study |
| **12** | Waiwai05 | EU095235 | Fagundes et al., 2008 |
| **13** | KTN18 | EU095238 | Fagundes et al., 2008 |
| **14** | Coreguaje 1-31 | EU095536 | Tamm et al., 2007 |

**Table S6.** Genbank and ID samples for D4h3a and D1 tree (figure S3).

**References**

Perego UA, Achilli A, Angerhofer N, Accetturo M, Pala M, et al. (2009) Distinctive Paleo-Indian migration routes from Beringia marked by two rare mtDNA haplogroups. Curr Biol. 19: 1-8. 1.

Achilli A, Perego UA, Bravi CM, Coble MD, Kong QP, et al. (2008) The phylogeny of the four pan-American MtDNA haplogroups: implications for evolutionary and disease studies. PLoS ONE. 3: e1764.

Fagundes N, Kanitz R, Eckert R, Valls ACS, Bogo MR, et al. (2008) Mitochondrial Population Genomics Supports a Single Pre-Clovis Origin with a Coastal Route for the Peopling of the Americas. Am J Hum Genet. 82: 583–592.

Tamm E, Kivisild T, Reidla M, Metspalu M, Glenn-Smith D, et al. (2007) Beringian standstill and spread of Native American founders. PLoS ONE 2: 1-6.
