# Supplementary material for: An Alternative Model for the Early Peopling of Southern South America Revealed by Analyses of Three Mitochondrial DNA Haplogroups
Source: PLoS One. 2012 Sep 10;7(9):e43486. doi: 10.1371/journal.pone.0043486 (PMC3438176; doi:10.1371/journal.pone.0043486)
Supplement: Supplemental Discussion S1 — Discussion about mutational rate used. (DOC) [file pone.0043486.s010.doc]

**Supplemental discussion**

***Selection of the mutation rate***

The choice of the mutation rate to use in evolutionary studies has been a very controversial topic, mainly due to the difference in dates obtained with different rates. Due to the lack of archeological material in North America from the end of the Pleistocene, the mutation rate utilized in genetic data to date the entry of Paleoindians to America and other population events is a very important matter of discussion.

According to the study of Ho et al. (2005) based on mitochondrial sequences, the mutation rate is variable over time, that is the relation between mutation rate (pedigree) and substitution rate (fixation) may be described by an exponential curve, which would thus produce an overestimation of divergence time for recent events. Based on this study, other authors, such as Ho & Larsson, (2006), Endicott et al., (2009), Henn et al., (2009) and Soares et al., (2009) have questioned, among other things, the dates which have usually been given for the populating of America, 20,000 – 30,000 years BP (Forster et al., 2004; Achilli et al., 2008; Fagundez et al., 2008). According to these authors, the variability found in Native Americans is not consistent with the pre-Clovis hypothesis, and the arrival in America would have been around 13,000 years BP. However, Emerson, (2007) used the same data as Ho et al., (2005) and did not find support for the model used, shedding doubt on the differences between short-term and long-term mutation rates found by Ho. In another study, Kemp et al., (2007), studying ancient DNA of an Alaskan esquelete dated at 10,300 years BP, found that the estimated mean evolutionary rate for HVRI was between 34% and 44%/site per myr, a rate which is closer to the pedigree values than to the phylogenetic rates and also provides evidence against Ho’s hypothesis.

Due to the high difference in mutational rates between studies, we performed calculations for TMRCA with four different rates, covering lower and higher rates; 24% mutations per site per myr (Santos et al., 2005), 30.2 % (Endicott & Ho, 2008), 34% (Kemp et al., 2007) and 45% (Howell et al., 2003), and we also use the rho calculator for the control region provided by Soares et al. (2009) to transform the networks’ rho to ages. The results (see Table S3) showed important differences between the estimated dates. For example, for our D1g the dates ranged from 10 kya with Howell’s rate to almost 27 kya with Soares’ rate. Since the most ancient archeological record for Patagonia is 14,500 years BP, a date of 20 kya for the origin of D1g is hard to support. Howell´s rate gave a plausible date for the appearance of D1g; however, his 45% mutational rate is often considered too high for demographic process date estimation. The dates from Kemp and Endicott are similar, with almost 1,500 years difference and both agree with the archeological register. Since Endicott´s rate was obtained for the complete D-loop, we used this rate for the Bayesian Skyline Plot analysis and TMRCA principal date for our model analysis. In relation to the Soares´ rate, it should be the most suitable rate since it was calculated for the entire control region (16024–576), and compensates for the effects of purifying selection on mtDNA sequence evolution. However, with this rate we obtained an extremely high age (27 Kyr), out of range of the founder Amerindian lineages origins. Soares et al. (2009) reported a rate of 9.883x10^-8^ mutations per nucleotide per year or one mutation each 9058 years for the whole control region, which is closer to the phylogenetic rates than the pedigree rates, and also lower than the rates used in this study. So if we take the Soares mutational rate, the origin of D1g should be placed in Beringia (or even Asia), which is highly improbable, since no other lineage related to D1g has been found in America.

Our analyses were performed with the hypervariable region of mtDNA, which has been used in the majority of phylogeographic studies due to its high rate of change (Pakendorf & Stoneking, 2005). We are well aware that the use of this region has some drawbacks, such as having a greater quantity of hotspots than other regions of mtDNA (Soares et al., 2009). This produces substitutions without a phylogenetic relation, altering the phylogenies and overestimating divergence times, because of which some authors have suggested that this region should not be used. However, since the divergence times for the founding lineages in America are much less than those of Africa, Europe, Asia and Oceania (Hill et al., 2006; Richards et al., 2000; Derenko et al., 2007; Friedlaender et al., 2007; Atkinson et al., 2009); eliminating the hypervariable sites might have the undesirable effect of underestimating the variability and thus the processes of divergence. Due to the short mutational time which has occurred since the initial colonization (which in the case of Patagonia, using Monte Verde as reference, would be a maximum of 15,000 years), it is arguable whether the time since the origin of the lineages has been sufficient to fix variability specific to populations as has occurred in others parts of the world, like Africa or Europe. For this reason we decided not to eliminate homoplastic, unique or non-informative sites from the analyses, but to keep in mind that this could produce overestimations of separation times, and so use a mutation rate concordant with the evolutionary process, in this case mutation rates closer to those of pedigrees than phylogenies, like the Endicott & Ho, (2008) rate.

**References**

1. Ho SY, Phillips MJ, Cooper A, Drummond AJ. (2005) Time dependency of molecular rate estimates and systematic overestimation of recent divergence times. Mol Biol Evol. 22:1561-1568.
2. Ho SY, Larson G. (2006) Molecular clocks: when times are a-changin’. Trends Genet. 22:79-83.
3. Endicott P, Ho SY, Metspalu M, Stringer C. (2009) Evaluating the mitochondrial timescale of human evolution. Trends Ecol Evol. 24:515-521.
4. Henn B M, Gignoux CR, Feldman MW, Mountain J. (2009) Characterizing the time dependency of human mitochondrial DNA mutation rate estimates. Mol Biol Evol. 26:217-230.
5. Soares P, Ermini L, Thomson N, Mormina M, Rito T, et al. (2009) Correcting for purifying selection: an improved human mitochondrial molecular clock. Am J Hum Genet. 84: 740–759.
6. Achilli A, Perego UA, Bravi CM, Coble MD, Kong QP, et al. (2008) The phylogeny of the four pan-American MtDNA haplogroups: implications for evolutionary and disease studies. PLoS One. 3: e1764.
7. Fagundes N, Kanitz R, Eckert R, Valls ACS, Bogo MR, et al. (2008) Mitochondrial Population Genomics Supports a Single Pre-Clovis Origin with a Coastal Route for the Peopling of the Americas. Am J Hum Genet. 82: 583–592.
8. Forster P. (2004) Ice Ages and the mitochondrial DNA chronology of human dispersals: a review. Phil Trans R Soc Lond B. 359: 255-264.
9. Emerson BC. (2007) Alarm bells for the molecular clock? No support for Ho et al.ʼs model of time-dependent molecular rate estimates. Syst Biol 56:337-45.
10. Kemp BM, Mahli RS, McDonough J, Bolnick DA, Eshleman JA, et al. (2007) Genetic analysis of early Holocene skeletal remains from Alaska and its implications for the settlement of the Americas. Am J Phys Anthropol. 132:605–621.
11. Santos C, Montiel R, Sierra B, Bettencourt C, Fernandez E, et al. (2005) Understanding differences between phylogenetic and pedigree-derived mtDNA mutation rate: a model using families from the Azores Islands (Portugal). Mol Biol Evol. 22:1490-505.
12. Endicott P, Ho SYW. (2008) A Bayesian Evaluation of Human Mitochondrial Substitution Rates. Am J Hum Genet. 82:895-902.
13. Howell N, Smejkal CB, Mackey DA, Chinnery PF, Turnbull DM, et al. (2003) The pedigree rate of sequence divergence in the human mitochondrial genome: There is a difference between phylogenetic and pedigree rates. Am J Hum Genet 72:659-670.
14. Pakendorf B, Stoneking M. (2005) Mitochondrial DNA and human evolution. Annu Rev Genomics Hum Genet. 6:165-83.
15. Hill C, Soares P, Mormina M, Macaulay V, Meehan W, et al. (2006) Phylogeography and ethnogenesis of aboriginal Southeast Asians. Mol Biol Evol. 23:2480-91.
16. Richards M, Macaulay V, Hickey E, Vega E, Sykes B, et al. (2000) Tracing European founder lineages in the Near Eastern mtDNA pool. Am J Hum Genet. 67:1251-76.
17. Derenko M, Malyarchuk B, Grzybowski T, Denisova G, Dambueva I, et al. (2007) Phylogeographic analysis of mitochondrial DNA in northern Asian populations. Am J Hum Genet. 81:1025-41.
18. Friedlaender JS, Friedlaender FR, Hodgson JA, Stoltz M, Koki G, et al. (2007) Melanesian mtDNA complexity. PLoS One. 2:e248.
19. Atkinson QD, Gray RD, Drummond AJ. (2009) Bayesian coalescent inference of major human mitochondrial DNA haplogroup expansions in Africa. Proc Biol Sci. 276:367-73.
