# Supplementary material for: An Alternative Model for the Early Peopling of Southern South America Revealed by Analyses of Three Mitochondrial DNA Haplogroups
Source: PLoS One. 2012 Sep 10;7(9):e43486. doi: 10.1371/journal.pone.0043486 (PMC3438176; doi:10.1371/journal.pone.0043486)
Supplement: Supplemental Networks S1 — Network made by hand for mitochondrial haplogroups A2, B2, D1, C1b and D4h3. The numbers correspond to the table S5. (PPT) [file pone.0043486.s011.ppt]

## Slide 1
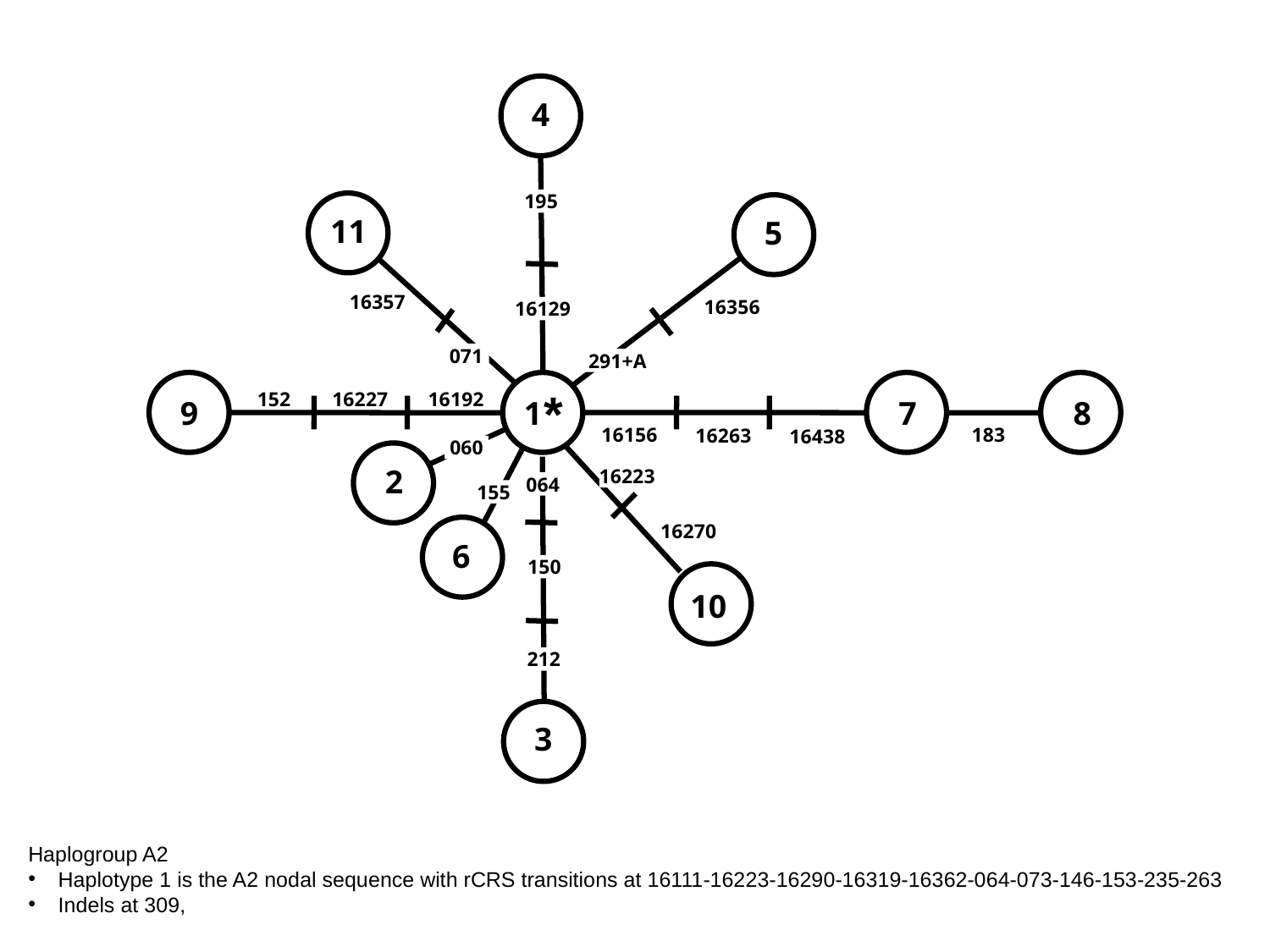

4
195
11
5
16357
16356
16129
071
291+A
*
152
16227
16192
9
1
7
8
16156
183
16263
16438
060
2
16223
064
155
16270
6
150
10
212
3
Haplogroup A2
Haplotype 1 is the A2 nodal sequence with rCRS transitions at 16111-16223-16290-16319-16362-064-073-146-153-235-263
Indels at 309,

## Slide 2
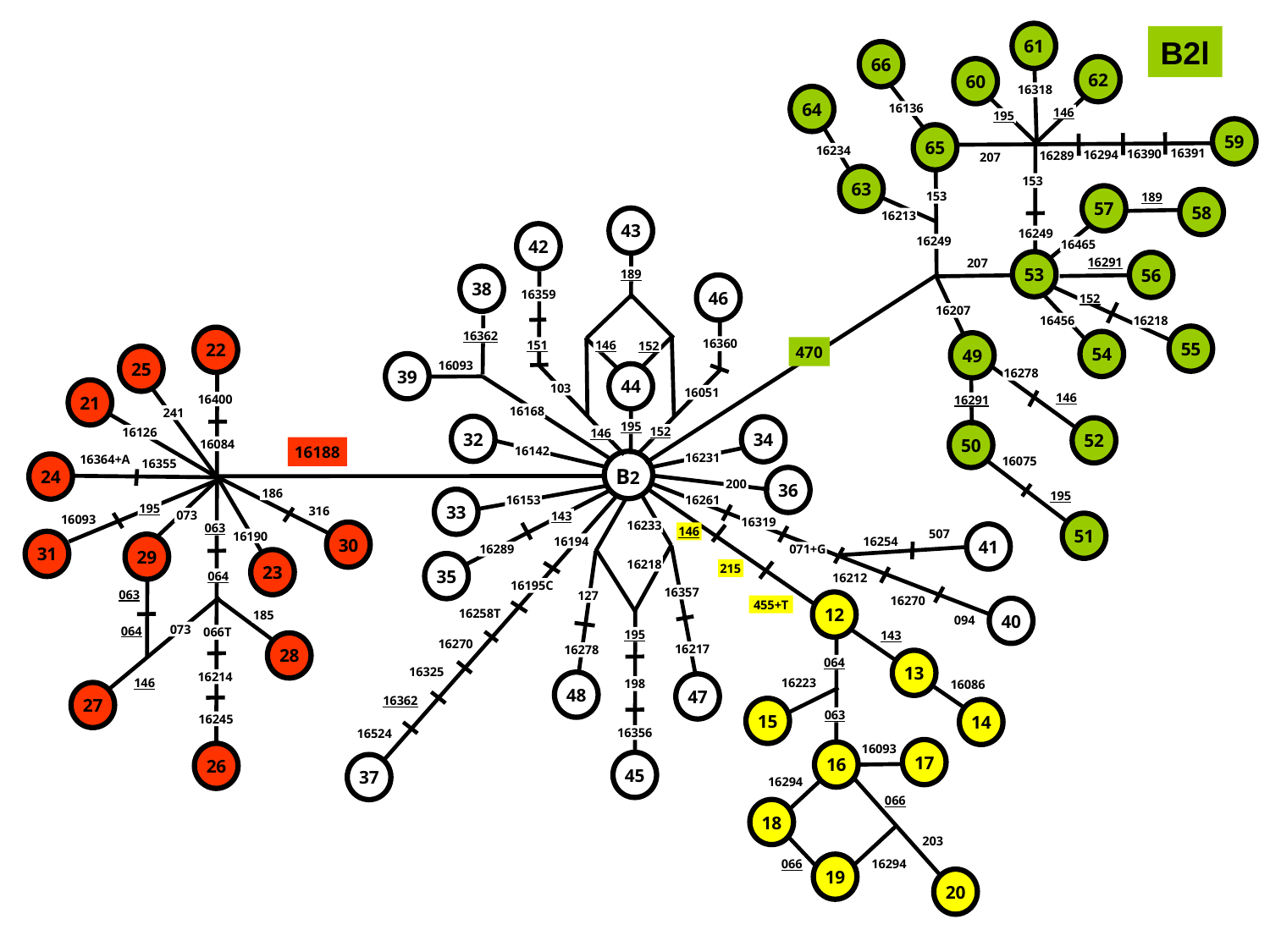

61
B2l
66
62
60
16318
64
16136
146
195
59
65
16234
16391
16390
16294
16289
207
63
153
57
153
58
189
16213
43
42
16249
16249
16465
53
56
16291
207
38
189
46
16359
152
16207
16456
16218
55
22
16362
54
49
16360
151
470
146
152
25
39
16093
44
16278
21
103
16051
146
16400
16291
16168
241
32
34
52
195
50
152
16126
146
16084
16188
16142
16231
16364+A
16075
24
16355
B2
36
200
186
195
33
16261
16153
195
316
073
143
16093
51
16319
16233
063
30
146
41
507
16190
31
16194
16254
29
16289
071+G
23
35
16218
215
215
064
16212
16195C
16357
063
127
12
16270
455+T
40
16258T
185
094
073
064
066T
195
143
28
16270
16217
16278
13
064
16325
16214
48
47
146
16223
198
16086
27
16362
15
14
063
16245
16356
16524
17
16093
16
26
45
37
16294
066
18
203
19
16294
066
20

## Slide 3
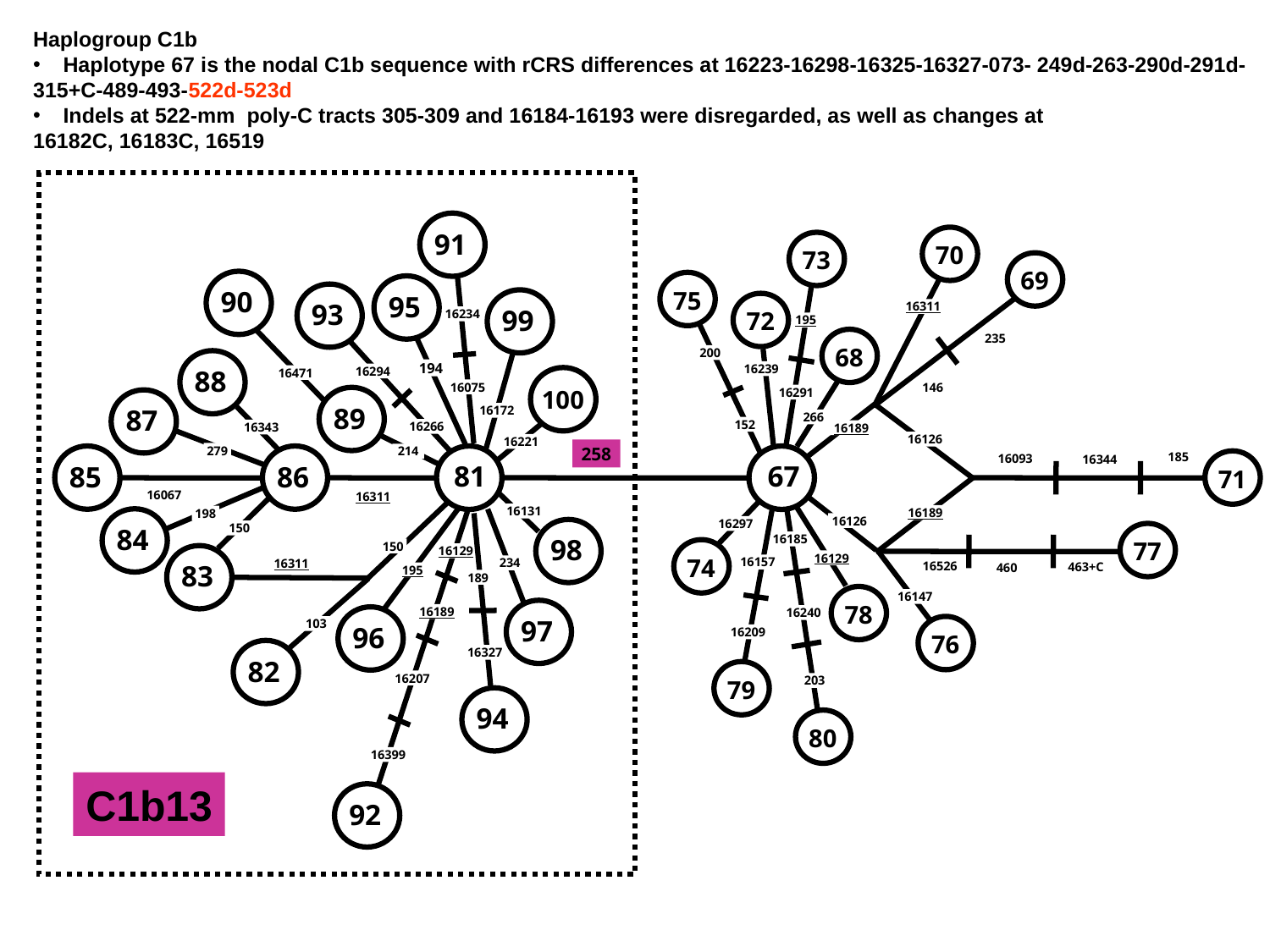

Haplogroup C1b
Haplotype 67 is the nodal C1b sequence with rCRS differences at 16223-16298-16325-16327-073- 249d-263-290d-291d-
315+C-489-493-522d-523d
Indels at 522-mm poly-C tracts 305-309 and 16184-16193 were disregarded, as well as changes at
16182C, 16183C, 16519
91
70
73
69
90
75
95
93
99
72
16311
16234
195
68
235
200
88
194
16239
16294
16471
16075
146
100
16291
89
87
16172
266
152
16266
16343
16189
16126
16221
258
279
214
85
86
185
16093
71
16344
81
67
16067
16311
16131
16189
198
84
16126
16297
98
150
77
16185
150
74
16129
83
16129
16157
234
16311
16526
463+C
460
195
189
78
16147
97
16189
16240
96
103
76
16209
82
16327
79
16207
203
94
80
16399
C1b13
92

## Slide 4
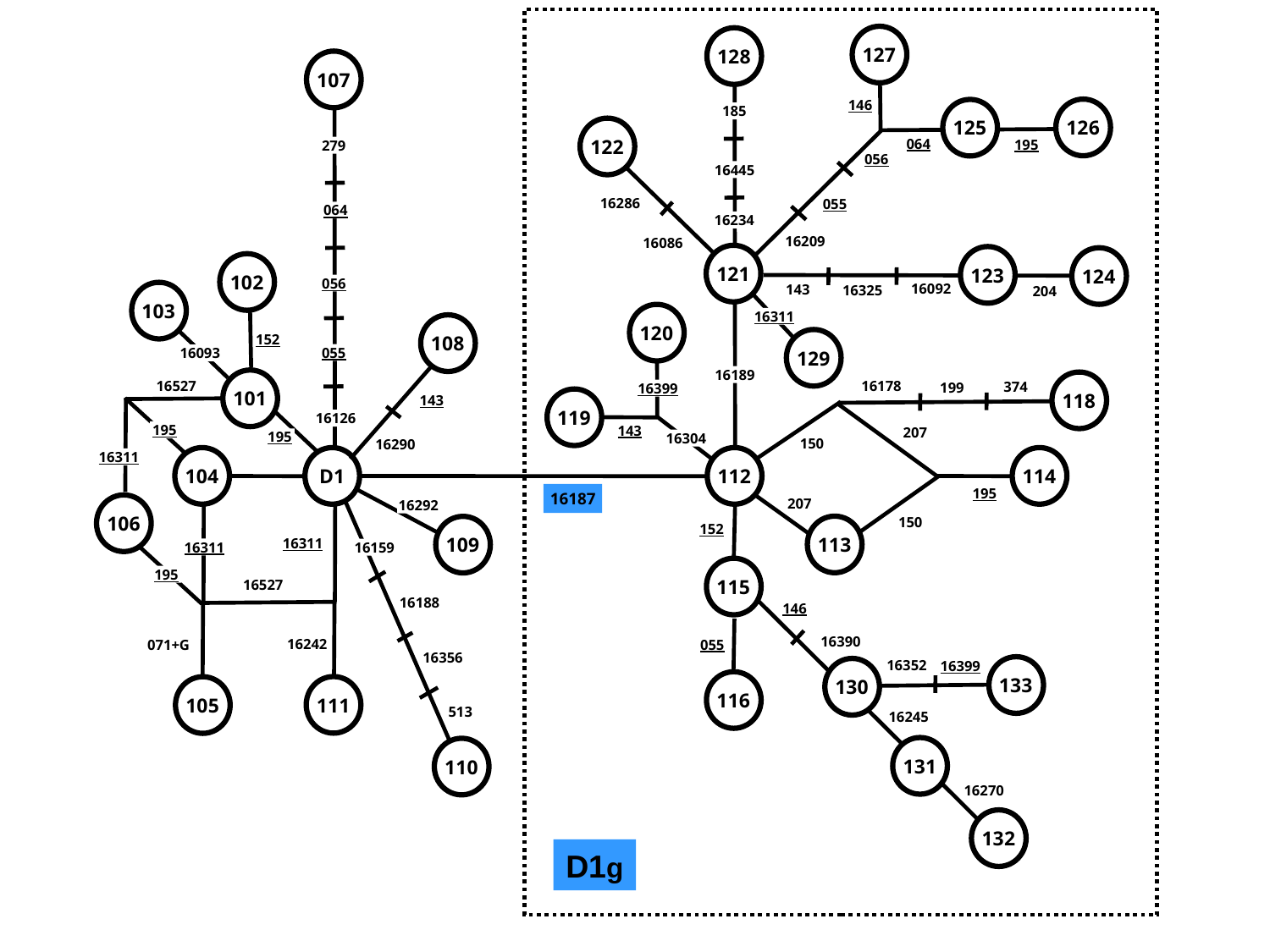

127
128
107
146
126
125
185
122
064
195
279
056
16445
16286
055
064
16234
16209
16086
121
123
124
102
056
16092
143
16325
103
204
120
16311
108
129
152
055
16093
16189
101
118
16527
16178
374
199
16399
119
143
16126
195
143
207
195
16304
150
16290
104
D1
112
114
16311
16187
195
106
207
16292
150
113
109
152
16311
16311
16159
115
195
16527
16188
146
16390
16242
071+G
055
16356
133
16352
16399
130
116
111
105
513
16245
131
110
16270
132
D1g

## Slide 5
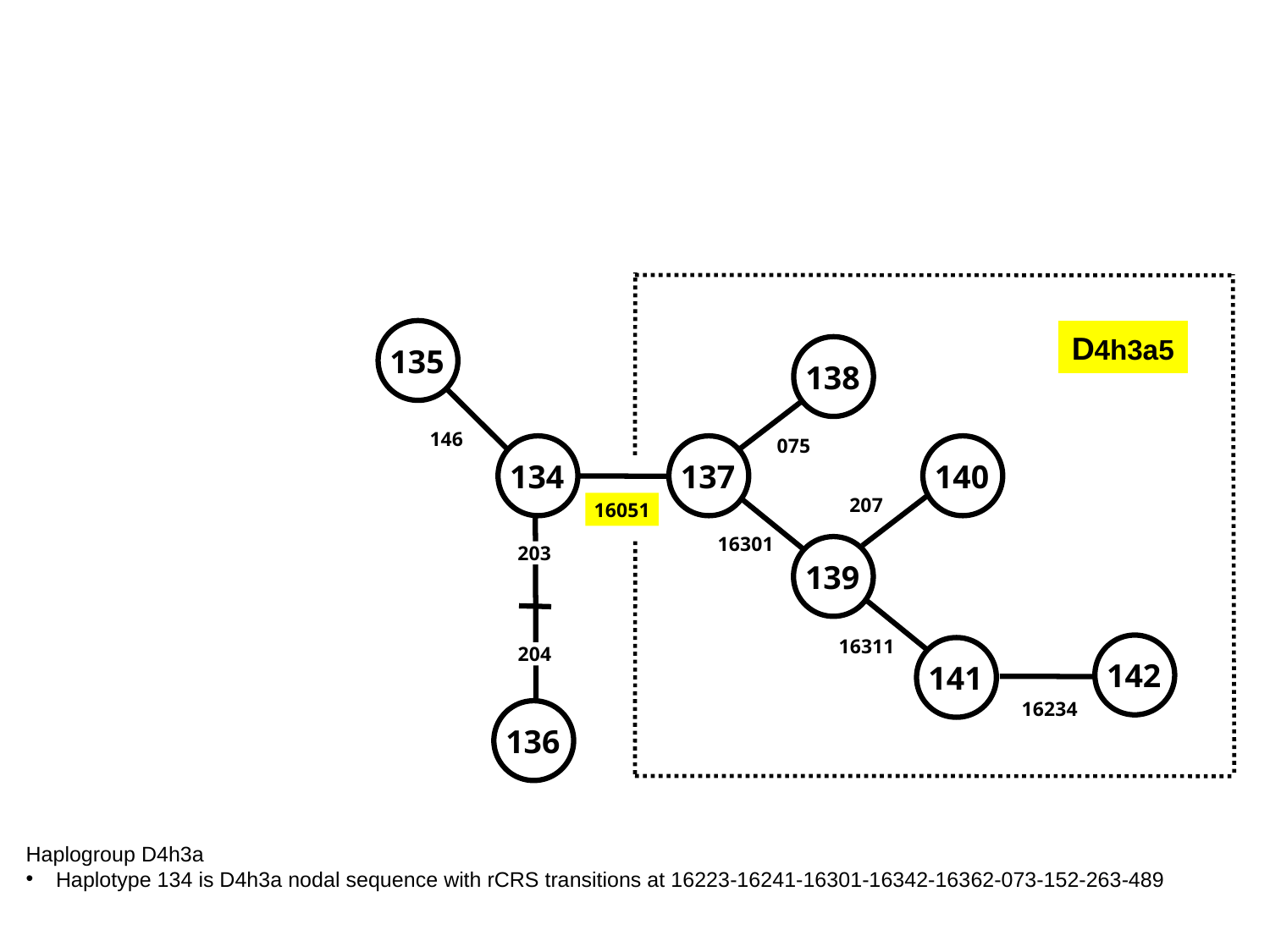

135
D4h3a5
138
146
075
134
137
140
16051
207
16301
139
203
16311
142
141
204
16234
136
Haplogroup D4h3a
Haplotype 134 is D4h3a nodal sequence with rCRS transitions at 16223-16241-16301-16342-16362-073-152-263-489
